# Generation and Characterization of a TRIM21 Overexpressing Mouse Line

**DOI:** 10.1002/dvg.23616

**Published:** 2024-11-01

**Authors:** Lisa M. Mehlmann, Tracy F. Uliasz, Siu‐Pok Yee, Deborah Kaback, Katie M. Lowther

**Affiliations:** ^1^ Department of Cell Biology UConn Health Farmington Connecticut USA

**Keywords:** mouse, oocyte, protein degradation, TRIM21, Trim‐Away

## Abstract

Specific removal of a protein is a key to understanding its function. “Trim‐Away” utilizes TRIM21, an antibody receptor and ubiquitin ligase, for acute and specific reduction of proteins. When TRIM21 is expressed in cells, introduction of a specific antibody causes rapid degradation of the targeted protein; however, TRIM21 is endogenously expressed in few cell types. We have generated a mouse line using CRISPR to insert a conditional overexpression cassette of TRIM21 into the safe harbor site, *Rosa26*. These conditionally‐expressing mice can be bred to a wide variety of *Cre* mice to target cell‐specific TRIM21 overexpression in different tissues. *Zp3* ^*Cre*^ mice expressed TRIM21 protein specifically in oocytes, whereas *Hprt* ^*Cre*^ mice expressed the protein globally. When TRIM21‐overexpressing oocytes were microinjected with specific antibodies targeting either the IP_3_ receptor or SNAP23, these proteins were effectively degraded. In addition, cortical neural cells from globally‐overexpressing TRIM21 mice showed a dramatic reduction in IP_3_ receptor protein within hours after electroporation of a specific antibody. These experiments confirm the effectiveness of the Trim‐Away method for protein reduction. These mice should make a valuable addition to the broader research community, as a wide range of proteins and cell types can be studied using this method.

## 1 Introduction

Inhibiting a protein's function is key to understanding its role in any given biological process. There are several methods for inhibiting protein function: pharmacological inhibition; application of antibodies that block protein function; gene knockouts, which prevent a gene of interest from being transcribed; RNA interference (RNAi) or antisense RNA, which specifically deplete RNA with the expectation that this will lead to a reduction in protein; CRISPR/Cas13 gene silencing (Powell et al. 2022); and application of morpholinos, which disrupt protein translation. While each of these methods has proven to be valuable, there are disadvantages of each. Pharmacological inhibitors are often nonspecific and can lead to off‐target effects. The effectiveness of RNA depletion methods, gene silencing, or use of morpholinos depends on protein stability, with very stable proteins being resistant to depletion. In addition, gene knockouts, RNAi, and morpholinos are chronic alterations that are often accompanied by compensatory mechanisms that can mitigate their effect.

**FIGURE 1:**
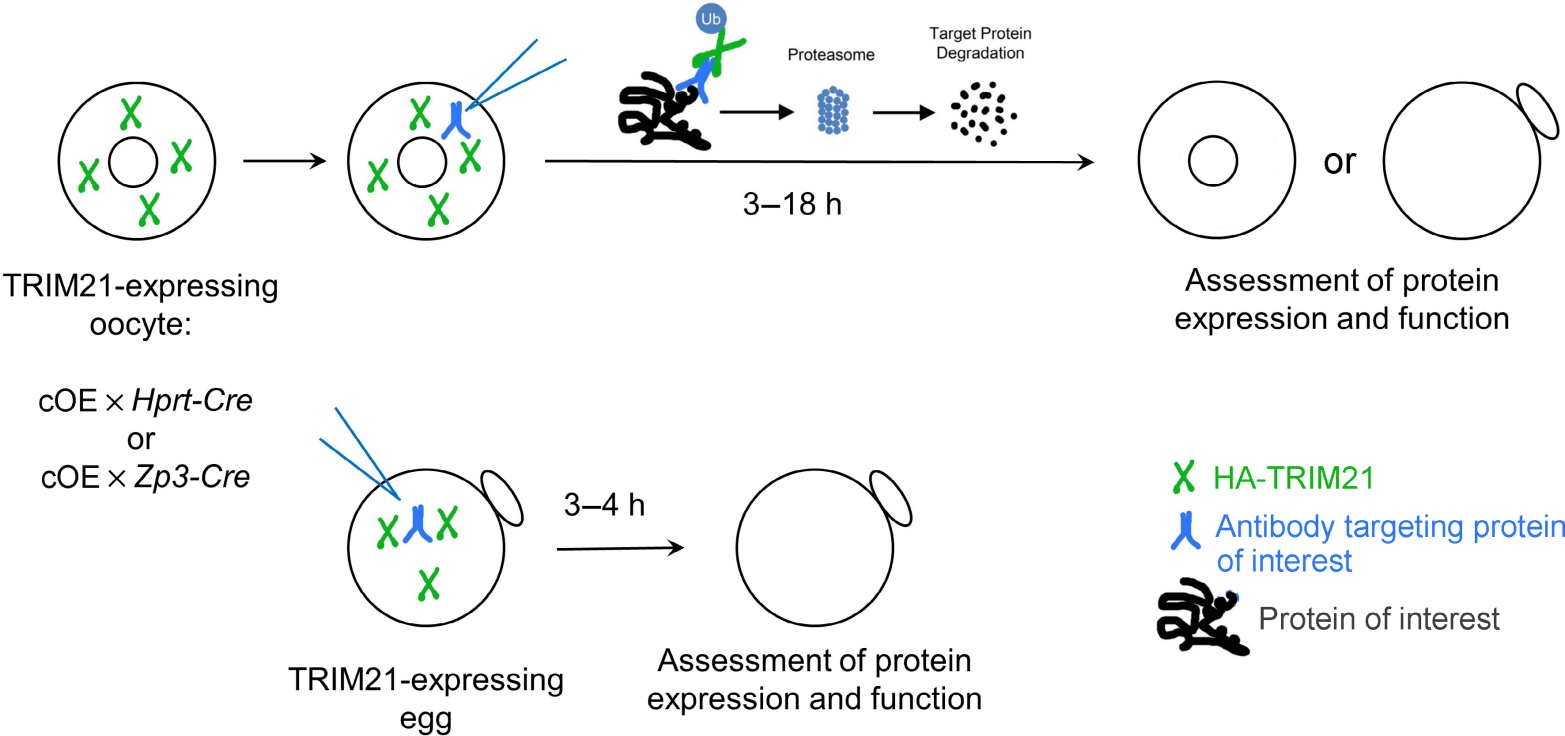
Mechanism of Trim‐Away method and experimental design for oocyte experiments. cOE = mice that contain the conditional overexpression cassette with CAG promoter allele.

A new method for rapid and acute protein depletion was introduced recently and used successfully in a wide range of cell types (Clift et al. 2017). This method, known as “Trim‐Away,” takes advantage of the protein TRIM21, which is either endogenously expressed in cells or is introduced by microinjecting or transfecting mRNA or electroporating purified protein. TRIM21 is an intracellular antibody receptor and ubiquitin ligase that binds to the Fc portion of antibodies (Mallery et al. 2010). When the antibody is bound to a specific protein, TRIM21 self‐ubiquitinates and the complex is shuttled into the proteasome pathway and degraded (Mallery et al. 2010). In practice, once TRIM21 protein is expressed in cells, specific antibodies targeting the desired protein for depletion are introduced by microinjection or electroporation. The antibody binds to the protein, TRIM21 binds to the antibody, and the complex is degraded by the proteasome (Figure 1). This method has been used in cultured mammalian cells and terminally‐differentiated cells such as mouse oocytes and neurons (Clift et al. 2017). While TRIM21 is endogenously expressed in several cell types, particularly in immune cells (Yoshimi et al. 2009), other cells, such as mouse oocytes, do not express it and the protein and antibodies must both be microinjected into mouse oocytes, either in combination or sequentially, although TRIM21 can be stably expressed in cultured cells (Clift et al. 2017).

Trim‐Away is an especially valuable method for protein depletion in cells that express stable proteins, such as oocytes. Many proteins in oocytes can be difficult to knock down using other common methods such as RNAi because of the relatively short lifespan (~2 days) of oocytes cultured in vitro (Mehlmann 2005). Mature, MII‐stage oocytes (herein referred to as “eggs”) have an even shorter lifespan (< 24 h; (Lacham‐Kaplan and Trounson 2008)). While it is possible to introduce TRIM21 and antibodies into immature oocytes and mature them to eggs in vitro such that the protein to be depleted is degraded prior to or during oocyte maturation (Mehlmann, Uliasz, and Lowther 2019), this method has disadvantages: (1) if the protein is necessary for oocyte maturation, the oocytes will not mature to metaphase II (MII); and (2) maturation of oocytes in vitro yields eggs that have lower developmental potential compared to oocytes matured in vivo (Lacham‐Kaplan and Trounson 2008). RNA encoding TRIM21 and the desired antibody can be co‐introduced into mature, freshly ovulated eggs by microinjection, but it takes hours for TRIM21 to be expressed, during which time the eggs are aging. For these reasons, it would be beneficial to have a mouse that constitutively expresses TRIM21 protein in oocytes and ovulated eggs. Here, we report the generation and characterization of such a mouse line. The main focus is on mouse oocytes and eggs, but tissue‐specific expression of TRIM21 can be achieved by breeding TRIM21‐positive founders with mice expressing tissue‐specific *Cre* recombinase.

## 2 Results and Discussion

### 2.1 Generation of HA‐TRIM21 Conditionally‐Overexpressing Mice

**FIGURE 2:**
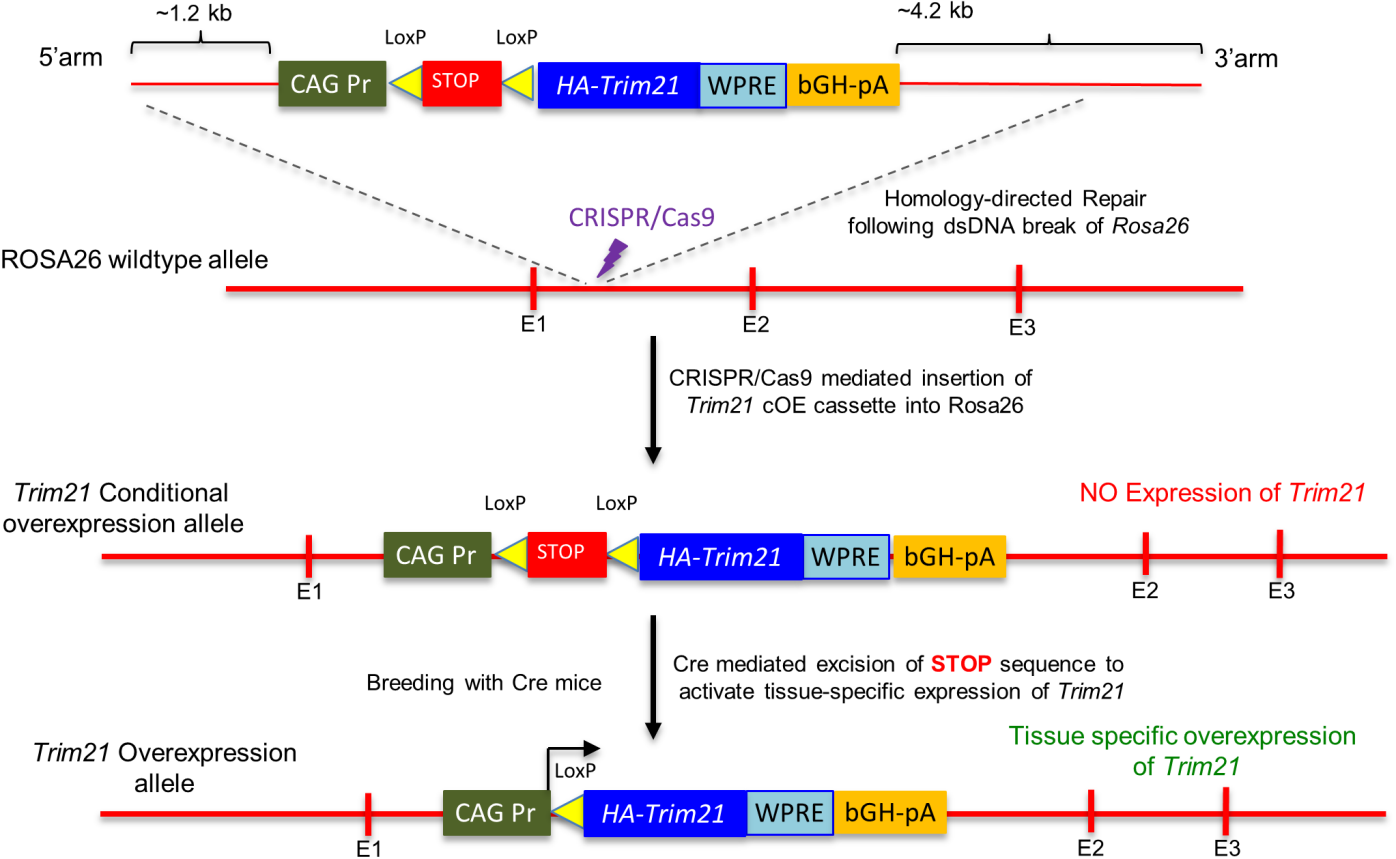
Strategy for production of TRIM21 conditional overexpression knock‐in mice. The targeting vector contains the conditional overexpression (cOE) cassette with CAG promoter (green box) followed by a LoxP‐STOP‐LoxP sequence (yellow triangles flanking the red box), *HA‐Trim21* coding sequence (dark blue box), a woodchuck hepatitis virus posttranscriptional regulatory element (WPRE; light blue box), and a bovine growth hormone polyadenylation signal sequence (bGH‐pA; orange box). This cassette is flanked by 1.2 and 4.2 kb of 5′ and 3′ homologous arms, respectively. The stop sequence is a 3× SV40 polyadenylation signal sequence and prevents *Trim21* expression from the cOE allele. After crossing the cOE mice to *Hprt* ^*Cre*^ or *Zp3* ^*Cre*^, the stop sequence is removed and the CAG promoter drives the expression of *HA‐Trim21* in cells expressing *Cre* recombinase (black arrow).

HA‐tagged *Trim21* conditionally‐overexpressing mice were generated in conjunction with the Center for Mouse Genome Modification (CMGM) at UConn Health using CRISPR/CAS9‐mediated gene editing to insert a *Trim21* conditional overexpression cassette into intron 1 of *Rosa26*. Design of the *Rosa26* targeting vector was similar to that of Ai9 (Addgene #22799), which was used to generate a *Cre*‐dependent tdTomato reporter mouse line (Madisen et al. 2010). The *Rosa26* locus is considered a safe harbor site that has been used for targeted overexpression of numerous genes without any undesirable phenotypes (Chu et al. 2016). The conditional overexpression cassette contains a CAG promoter (Niwa, Yamamura, and Miyazaki 1991) followed by a LoxP‐3xSV40 polyadenylation signal‐LoxP sequence, ~1.5 kb of *HA‐Trim21* coding sequence, a woodchuck hepatitis virus posttranscriptional regulatory element, and a bovine growth hormone polyadenylation signal sequence. This cassette is flanked by 1.2 and 4.2 kb of 5′ and 3′ homologous arms, respectively, aimed for homology‐directed repair after CRISPR/CAS9‐mediated double‐strand DNA break of the targeted site (Figure 2).

**FIGURE 3:**
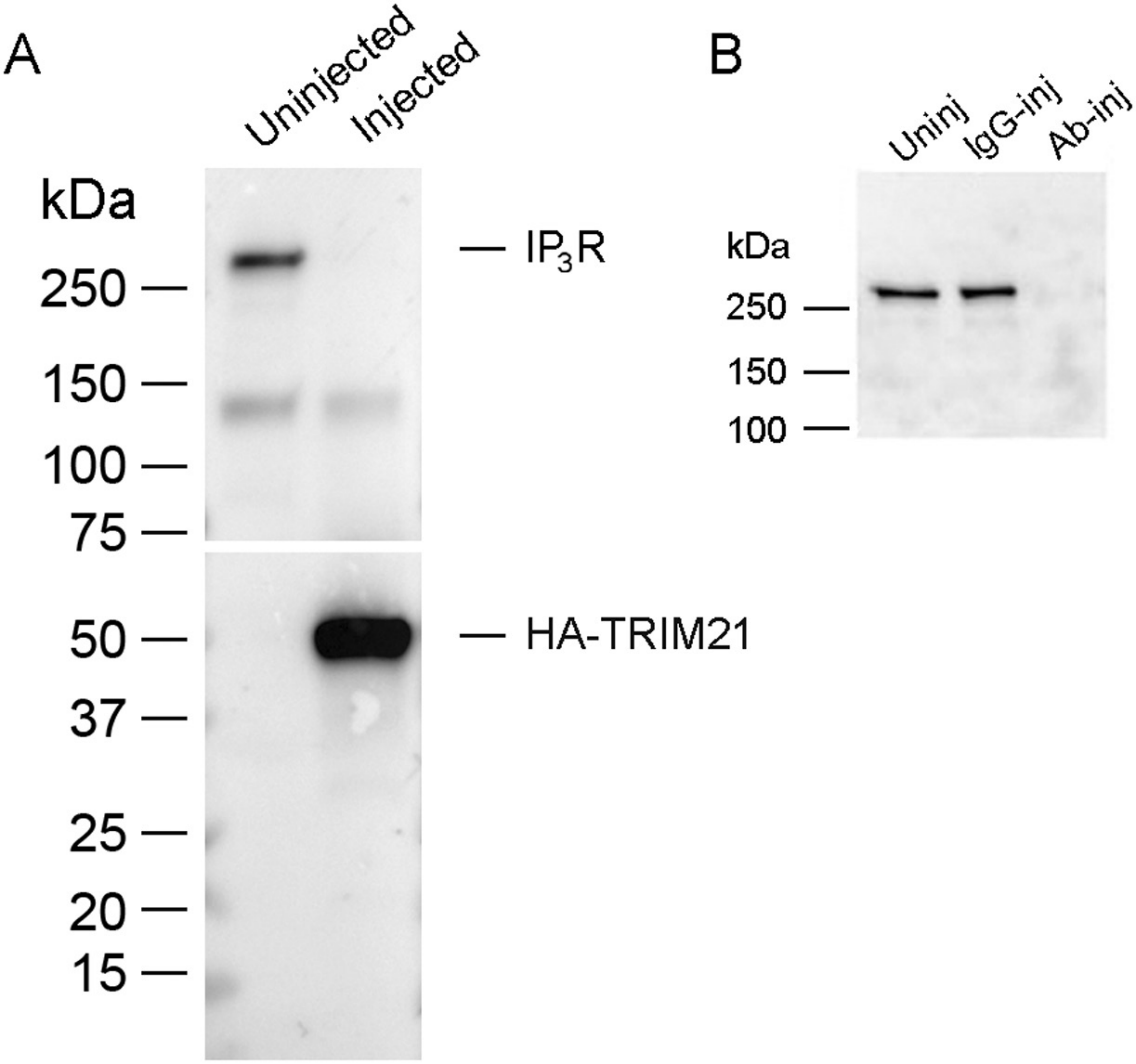
(A) The targeting vector used to construct the mouse line was functional, based on the overexpression of HA‐TRIM21 protein and the specific reduction in IP_3_R protein after microinjection into mouse oocytes. Lysates from 13 oocytes were run in each lane. (B) Trim‐Away reduces IP_3_R protein in TRIM21‐expressing oocytes, whereas injecting IgG has no effect on IP_3_R protein levels. Equal numbers of uninjected and injected oocytes were run in each lane.

Prior to generating the mouse model, we tested the expression and function of HA‐TRIM21 by microinjection of in vitro‐transcribed mRNA encoding ~1.5 kb of the HA‐TRIM21 coding sequence into mouse oocytes in conjunction with an antibody specifically against the inositol 1,4,5‐trisphosphate receptor (IP_3_R; (Runft, Watras, and Jaffe 1999)). We chose this protein because it is a large, transmembrane protein expressed in mouse oocytes and eggs for which we have an antibody that permits its detection in just a few cells (Mehlmann, Mikoshiba, and Kline 1996). Microinjected GV‐stage oocytes were matured in vitro overnight and lysed in sample buffer after confirming that they had undergone germinal vesicle breakdown (GVBD) and extruded first polar bodies, indicating that they were in MII. A western blot probed with antibodies against the IP_3_R and the HA tag confirmed that the TRIM21 protein was synthesized and that the IP_3_R was robustly degraded (Figure 3A). Specificity of Trim‐Away in these oocytes was demonstrated by injecting nonimmune IgG into TRIM21‐expressing oocytes; IgG had no effect on IP_3_R levels (Figure 3B). After confirming that this vector produced functional TRIM21 protein in oocytes, the targeting vector and sgRNA/CAS9 ribonucleoprotein complex were microinjected into the pronuclei of one‐cell embryos derived from C57BL/6J mice. Following microinjection, embryos were transferred to recipient pseudopregnant females.

Potential founder mice were initially identified using primers specific to *Trim21* and the WPRE. The genotypes of positive mice were further analyzed by long‐range PCR to ensure that the cassette was properly inserted into *Rosa26*. Genotyping primers are listed in Tables 1 and 2. Founders were bred with wildtype mice to establish the TRIM21 conditional overexpression line (cOE). The *Trim21* conditional line was then either bred with *Hprt* ^*Cre*^ mice (Jax stock #004302; (Tang et al. 2002)) to generate global TRIM21 expression (OE) or with *Zp3* ^*Cre*^ mice (Jax stock #003651; (de Vries et al. 2000)) for oocyte‐specific TRIM21 expression. The overexpressing mice were then backcrossed for two generations to mice of the CF1 strain. This strain was chosen because it is most often used for oocyte studies in our lab and mice from this strain produce high quality, optically clear oocytes that are well suited for microinjection and fluorescent imaging.

**TABLE 1:** *Rosa26* long‐range PCR confirmation of proper integration.

Left short arm
R26 I1F1 5′‐GCTAGGTAGGGGATCGGGACTC
AiCAGR1A 5′‐GGCGTTACTATGGGAACATACGTC
Followed by
R26 I1F2 5′‐CTTGGTGCGTTTGCGGGGATG
AiCAGR1A 5′‐GGCGTTACTATGGGAACATACGTC
PCR ≥ 1.2 kb
Right long arm
RGpA5F1 5′‐CCTCCTCTCCTGACTACTCCCAG
RosaE2R1 5′‐GATCTCGAAGACCTGTTGCTGCTCAG
Followed by
RBGpAf 5′‐CTCCCAGTCATAGCTGTCCCTC
nR26E2R2 5′‐GCCTTAAACAAGCACTGTCCTGTCC
PCR ≥ 4.7 kb

**TABLE 2:** Genotyping primers.

Primer name	Primer sequence 5′–3′	Amplicon size
Trim213F R26‐WHVER	CCAGTCCTCAGACCACCCTCCAC GCGTATCCACATAGCGTAAAAGGAGC	366 bp for cOE and OE allele
CAG3F Trim215R	GGGTTCGGCTTCTGGCGTGTG CCACCATGGGATCCAGGCAG	394 bp for OE allele 1.2 kb for cOE allele
R26‐TDF R26‐TDR	CTCTGCTGCCTCCTGGCTTCTGAG CTCCGAGGCGGATCACAAGC	325 bp for WT allele
olMR1084 olMR1085	GCGGTCTGGCAGTAAAAACTATC GTGAAACAGCATTGCTGTCACTT	100 bp for Generic Cre

### 2.2 Characterization of HA‐TRIM21 Mice

**FIGURE 4:**
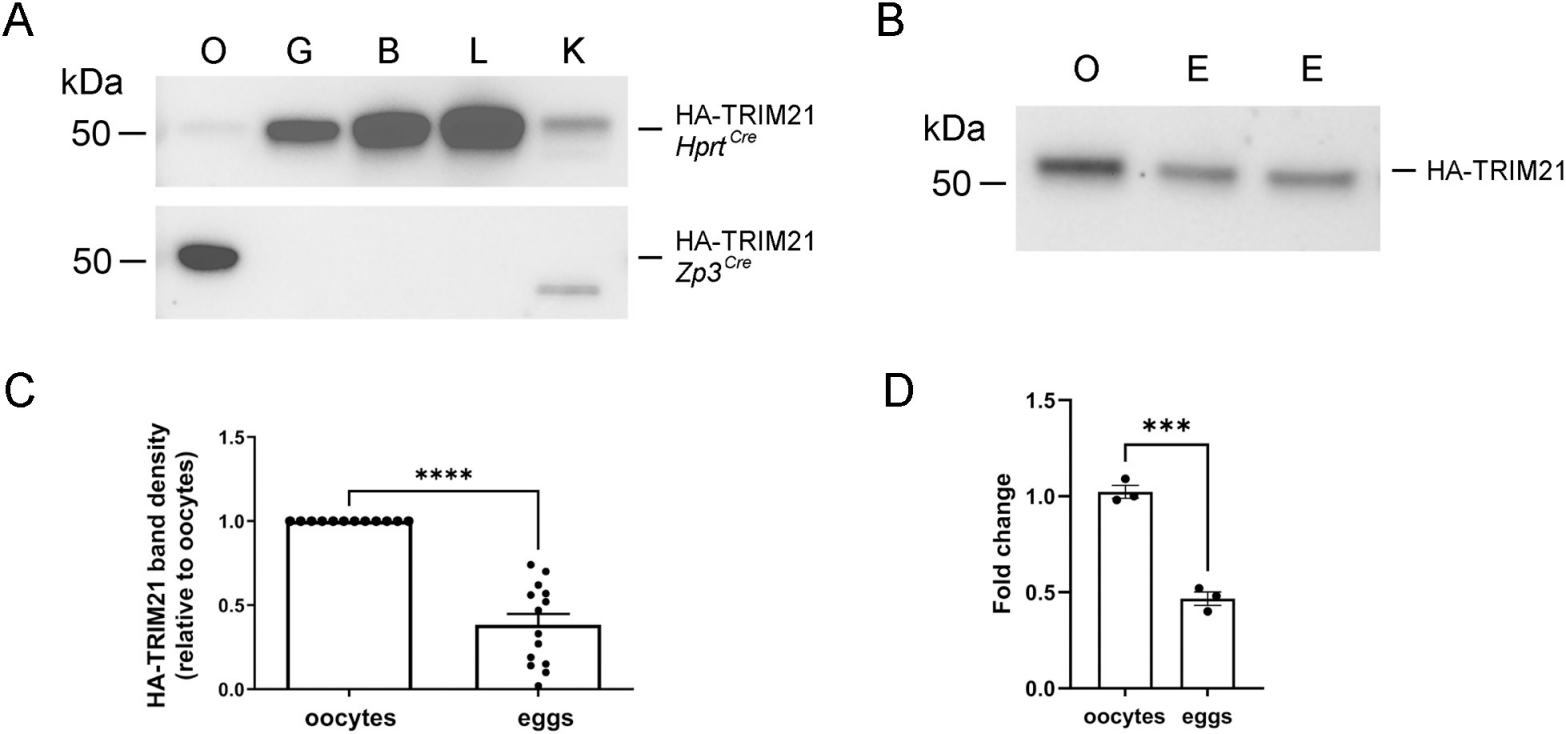
HA‐TRIM21 expression in oocytes, eggs, and other tissues. (A) Western blot showing HA‐TRIM21 protein in select tissues when cOE mice were bred with *Hprt* ^*Cre*^ mice (top panel) and specifically in oocytes when cOE mice were bred with *Zp3* ^*Cre*^ mice (bottom panel). O = oocytes, G = granulosa cells, B = brain, L = liver, K = kidney. One microgram oocyte and tissue lysates were run per lane. Blots are representative of three separate experiments using three mice of each genotype. (B) Western blot of lysate from 14 immature, GV‐stage oocytes and 14 mature, MII‐stage eggs. Oocytes and eggs were obtained from the same oocyte‐specific expressing HA‐TRIM21 (*Zp3* ^*Cre*^) mouse. (C) Quantification of HA‐TRIM21 immunoblot signal comparing the amount of HA‐TRIM21 in oocytes compared to eggs. Number of experiments is indicated in parentheses above each bar. The graph shows mean ± SEM; Paired *t*‐test; ****p < 0.0001. Data were obtained from *Hprt* ^*Cre*^ and *Zp3* ^*Cre*^ oocytes for the graph. (D) qPCR showing the relative levels of *Trim21* RNA expression in oocytes and eggs retrieved from *Zp3* ^*Cre*^ mice. Bars are mean ± SEM. Student's *t*‐test; ***p < 0.001.

Select tissues from offspring from *Hprt* ^*Cre*^ or *Zp3* ^*Cre*^ mice crossed with cOE mice were analyzed for expression of HA‐TRIM21 using western blot. Mice from *Hprt* ^*Cre*^ crosses showed HA‐TRIM21 expression in all tissues examined, including oocytes, granulosa cells, brain, liver, and kidney. Expression of HA‐TRIM21 in these tissues was variable, with granulosa cells, brain, and liver showing highest expression and oocytes and kidney having the lowest (Figure 4A, top panel). HA‐TRIM21 was abundantly expressed in oocytes from *Zp3* ^*Cre*^ crosses, with no expression in other tissues examined (Figure 4A, bottom panel). The amount of HA‐TRIM21 protein appeared to be significantly less in the oocytes from global knock‐in mice compared to the oocyte‐specific oocytes, although oocytes from these mice were not run on the same blot for comparison. Analysis of HA‐TRIM21 protein abundance showed that immature, GV‐stage oocytes that were still arrested in prophase contained about twice as much protein as mature, MII‐stage eggs obtained from the same mouse (Figure 4B,C). This decrease in expression could be at least in part due to *Trim21* RNA degradation during oocyte maturation, as we observed that the expression levels were ~50% lower in eggs than in oocytes (Figure 4D).

**FIGURE 5:**
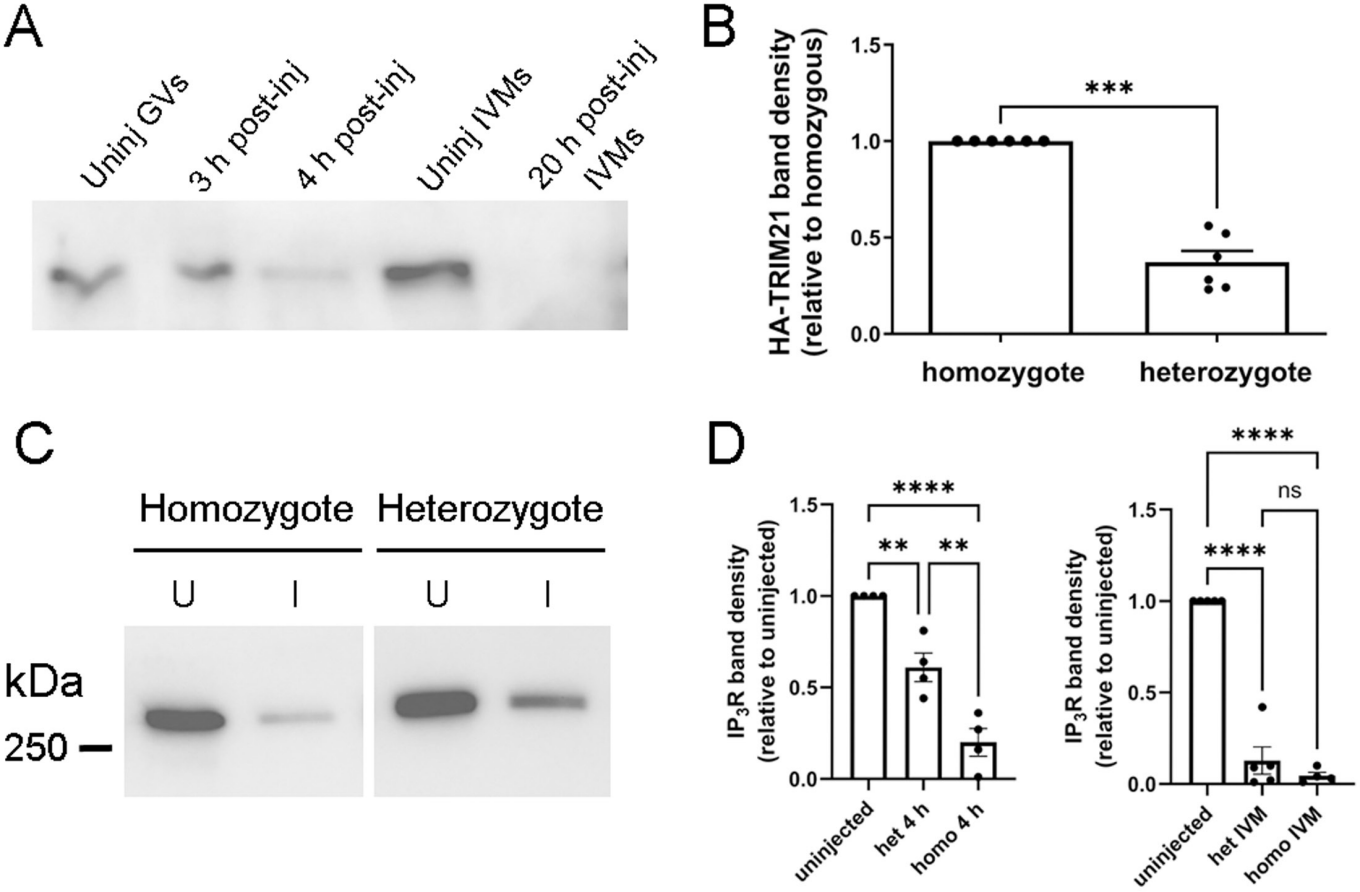
IP_3_R protein is degraded in HA‐TRIM21‐overexpressing oocytes following injection of a specific antibody against the IP_3_R. (A) Western blot showing protein degradation at 3 and 4 h after antibody injection, as well as after 20 h of in vitro maturation. Uninj GVs = uninjected, GV‐stage oocytes. 3‐ and 4‐h post‐inj = immature injected oocytes collected ~3 and 4 h after antibody injection. Uninj IVMs = uninjected oocytes matured overnight. 20 h post‐inj IVMs = injected oocytes matured overnight and collected ~20 h after injection. A total of six oocytes/eggs were run per lane. (B) Oocytes from homozygous mice contain approximately twice as much HA‐TRIM21 protein as heterozygous oocytes. The graph shows mean ± SEM of densitometric values taken from six separate experiments. Paired *t*‐test; ***p < 0.001. (C) Western blot showing that oocytes homozygous for HA‐TRIM21 more effectively degraded IP_3_R protein compared to heterozygotes. Equal numbers of oocytes were run in each lane. (D) Relative decrease in the amount of IP_3_R protein in heterozygous and homozygous oocytes that were injected with IP3R antibody and collected ~4 or ~18 h after injection. Bars are mean ± SEM. One‐way ANOVA; **p < 0.01; ****p < 0.0001; ns = not significant. Each experiment was performed at least three times.

To determine if endogenous levels of HA‐TRIM21 protein are sufficient for protein degradation, we first tested the ability of proteins to be degraded in oocytes heterozygous for *HA‐Trim21* when injected with a specific antibody targeting the IP_3_R. We microinjected IP_3_R antibody into immature oocytes and collected the oocytes 3 and 4 h postinjection, as well as after overnight in vitro maturation. We found a small decrease in IP_3_R protein 3 and 4 h after antibody injection, while the protein was undetectable in oocytes incubated overnight (Figure 5A). Because the time frame to achieve protein degradation to undetectable levels was much slower than degradation in oocytes in which HA‐TRIM21 is overexpressed by microinjecting mRNA (data not shown), we generated mice homozygous for *Trim21* with the assumption that the amount of TRIM21 protein would be higher in these oocytes. Indeed, the amount of HA‐TRIM21 was approximately twice as much in homozygotes compared to heterozygotes (Figure 5B). We then tested protein degradation in oocytes from mice homozygous for HA‐TRIM21 and found that 4 h after antibody injection, protein degradation was more robust in oocytes from homozygotes than from heterozygotes (Figure 5C), but the highest amount of degradation was still seen after overnight oocyte maturation (Figure 5D). These data suggest that while HA‐TRIM21 is constitutively expressed in oocytes, the amount is not sufficiently high to produce a complete loss of protein within 3–4 h.

One of the main purposes of creating an oocyte‐specific overexpressing TRIM21 mouse was to obtain ovulated, MII‐stage eggs that already contain TRIM21 protein. To this end, we superovulated mice homozygous for oocyte‐specific HA‐TRIM21 and microinjected ovulated eggs with antibodies against IP_3_R or SNAP23, a protein that we previously showed is essential for exocytosis in oocytes and eggs (Mehlmann, Uliasz, and Lowther 2019). For these experiments, we characterized the injected eggs ~4 h after antibody injection.

**FIGURE 6:**
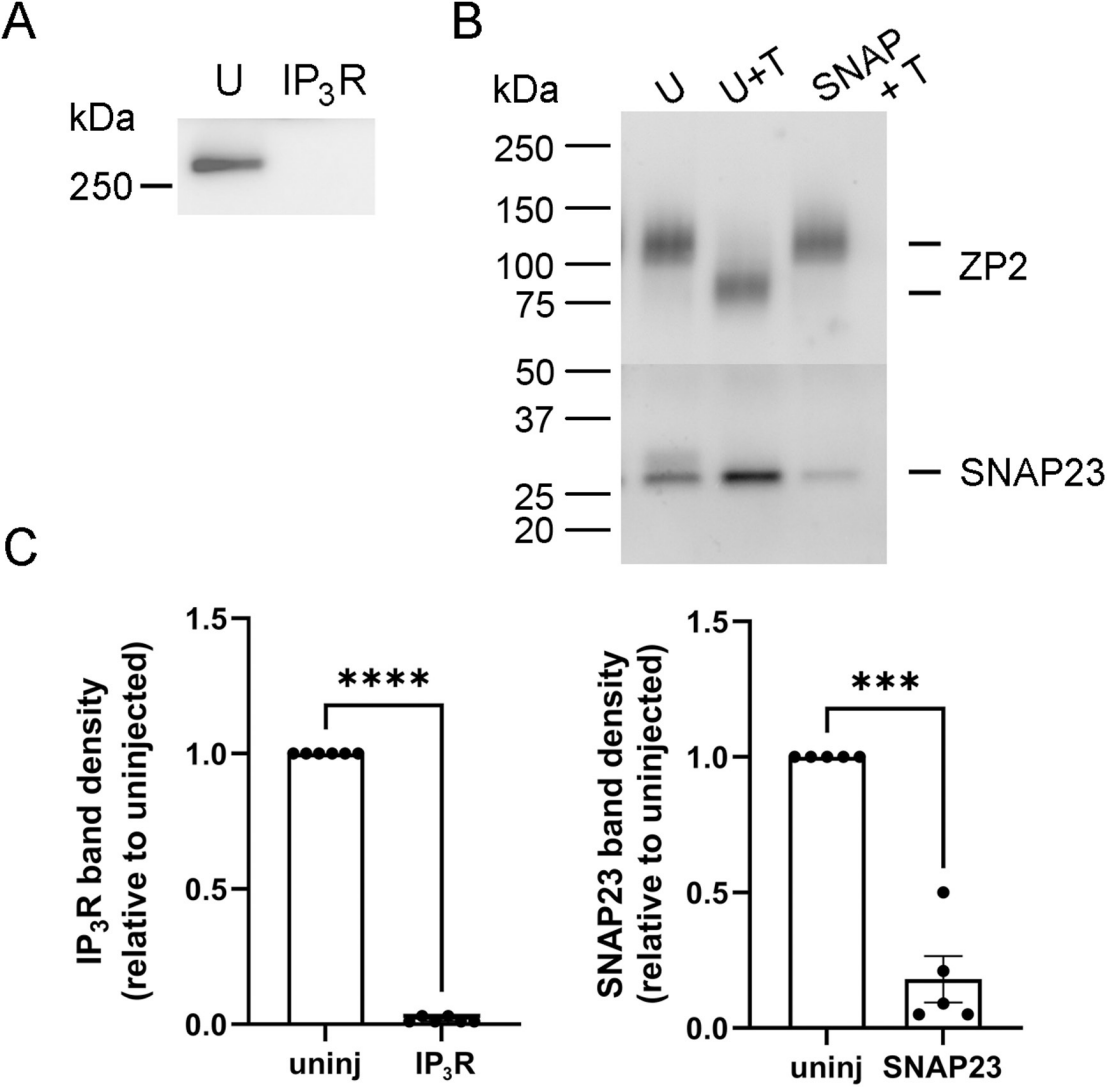
Effective protein degradation in ovulated, HA‐TRIM21‐expressing eggs following injection of specific antibodies. (A) Degradation of IP_3_R protein ~4 h after injection of a specific IP_3_R antibody. U = uninjected and IP_3_R = IP_3_R antibody‐injected eggs. Four uninjected and four injected eggs were run per lane. (B) Degradation of SNAP23 protein ~4 h after antibody injection was sufficient to prevent the cleavage of ZP2 after a Ca^2+^ stimulus. U = uninjected eggs; U + T = uninjected eggs treated with the Ca^2+^ releasing agent, thimerosal; SNAP + T = SNAP23 antibody‐injected eggs treated with thimerosal. A total of six eggs were run in each lane. (C) Relative decrease in IP_3_R and SNAP23 protein compared to uninjected oocytes. SNAP23 comparisons were made between thimerosal‐treated groups. Bars are mean ± SEM and each point represents one experiment. Paired *t*‐tests; ***p < 0.001; ****p < 0.0001.

IP_3_R protein was reduced to undetectable levels in homozygous eggs ~4 h after antibody injection (Figure 6A,C). SNAP23 protein was also effectively degraded within 4 h after antibody injection, although the protein was still detectable (Figure 6B,C). When SNAP23 antibody‐injected eggs were treated with thimerosal to stimulate cortical granule exocytosis 4 h after antibody injection (Mehlmann, Uliasz, and Lowther 2019), exocytosis was completely blocked as assessed by cleavage of the zona pellucida protein, ZP2 (Figure 6B). Normally, ZP2 is cleaved in response to a series of repetitive Ca^2+^ transients that are triggered by thimerosal treatment (Mehlmann and Kline 1994; Mehlmann, Uliasz, and Lowther 2019). This result shows that the loss of SNAP23 was sufficient to block its function and further demonstrates that these mice will be useful tools for studying the function of a wide variety of proteins in oocytes and eggs. It is unclear why we observed a more robust protein degradation response to antibody injection, and in a shorter time frame, than we found when injecting immature oocytes, particularly because it seems that some amount of TRIM21 protein is lost during oocyte maturation and mature eggs contain half as much protein as oocytes.

A possible limitation to this study is that the proteins we chose to deplete (IP_3_R and SNAP23), despite being detectable in just a few oocytes/eggs using western blot, are not highly expressed based on proteomics analysis (Jentoft et al. 2023). Degradation of more highly expressed proteins might be less complete, but effective protein depletion probably depends in large part on the particular antibody used.

### 2.3 Proteins in Other Cell Types Are Also Degraded in Mice Overexpressing HA‐TRIM21

**FIGURE 7:**
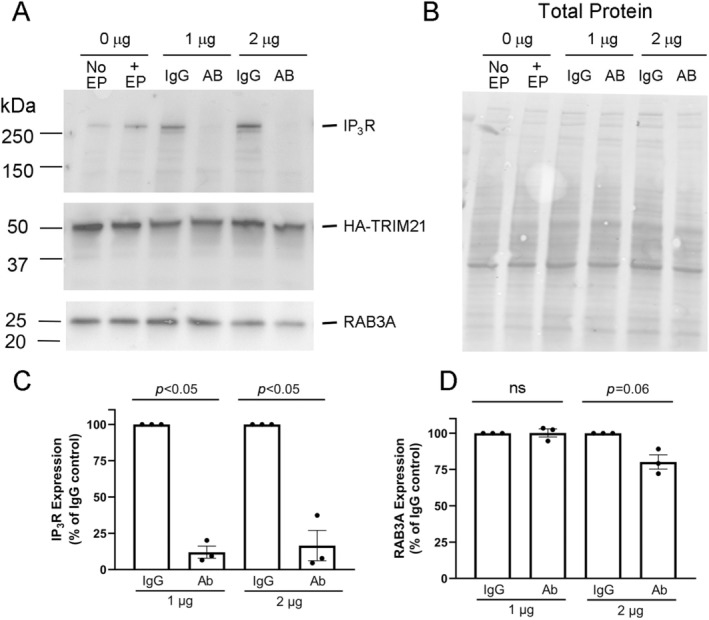
Effective degradation of IP_3_R in cortical cells from global‐expressing HA‐TRIM21 mouse brains. (A) TRIM21 protein is abundantly expressed in these cells (HA‐TRIM21, 0 μg bar) and IP_3_R is degraded in cells electroporated with 1 and 2 μg antibody but not in cells electroporated with control IgG. The amount of RAB3A did not decrease significantly in the electroporated cells. (B) Total protein stain showing equal loading in each lane. (C and D). Densitometric analysis of IP_3_R (C) and RAB3A (D) bands in electroporated cells. Bars are mean ± SEM and each point represents values from a single experiment. Paired *t*‐tests; ns = not significant.

To test the usefulness of these mice for studying other cell types, we used neuronal tissue. For these experiments, cortical brain tissue from global TRIM21 knock‐in mice was obtained from newborn mice and dissociated cells were cultured for 8–11 days. We introduced the same IP_3_R antibody we used in oocytes and eggs into neural cells using electroporation and collected the cells for western blot 4 h later. Cells that were not electroporated, or that were electroporated in the absence of IgG or antibody, abundantly expressed TRIM21 protein (Figure 7, No EP and +EP lanes). In three separate experiments, the amount of IP_3_R protein was significantly reduced after 4 h in cells electroporated with 1 and 2 μg antibody, whereas the protein was still expressed in cells electroporated with the same amount of IgG (Figure 7). To show the specificity of the protein depletion, we probed the blot with RAB3A antibody, as RAB3A is abundantly expressed in neuronal tissue (Fischer von Mollard et al. 1994). We did not detect a significant decrease in RAB3A protein in IP_3_R‐ compared to IgG‐electroporated cells (Figure 7). These experiments suggest that HA‐TRIM21‐overexpressing mice can readily be used to study protein function in many types of cells, making them of interest to the broader research community.

## 3 Materials and Methods

### 3.1 Plasmid Construction

A gBlock encoding the *HA‐Trim* sequence with unique 5′ and 3′ restriction sites was purchased from IDT. The gBlock was digested with SalI + SpeI for insertion into the pSK+ vector. The resulting vector “*HA‐Trim21‐SK*” was digested with NotI and used as a template for in vitro transcription using T7 polymerase. The *Rosa26* conditional overexpression vector was prepared as described previously (Hrdlicka et al. 2021). Briefly, the Ai9 plasmid (Addgene plasmid 22,799) was used as template for PCR and conventional cloning to prepare the *Rosa26* cOE vector with unique AsiSI restriction sites between the Lox‐STOP‐Lox and WPRE sequences. *HA‐Trim21‐SK* was digested with AsiSI, and the *HA‐Trim21* fragment (~1.5 kb) was gel purified and cloned into the AsiSI sites of the *Rosa26* cOE vector. Proper insertion of the *HA‐Trim21* sequence was initially determined by restriction digestion and correct directionality of the coding sequence was further confirmed by sequencing.

### 3.2 Mice

Animal studies were performed in accordance with the Guide for the Care and Use of Laboratory Animals (National Academy of Sciences 1996) and were approved by the Institutional Animal Care and Use Committee at UConn Health (protocol #101977‐0122). C57BL/6J female and male mice from The Jackson Laboratory (stock #000664) were used for pronuclear injection of the targeting vector and CAS9/sgRNA ribonucleoprotein complex (100 ng/μL CAS9 protein, 50 ng/μL crRNA, 100 ng/μL tracrRNA, 20 ng/μL targeting vector). The sequence of CRISPR site specific for *Rosa26* intron 1, 5′‐GAA GAT GGG CGG GAG TCT TC, was identified by http://chopchop.cbu.uib.no. CAS9 protein was purchased from Sigma and crRNA and tracrRNA were purchased from IDT. One‐cell embryos from the microinjection were transferred the same day to oviducts of CD‐1 (Charles River CRL:CD1(ICR)) pseudopregnant recipients. To achieve global‐ or oocyte‐specific overexpression, mice were then bred to either *Hprt* ^*Cre*^ mice (Jax stock #004302) that had been backcrossed with C57BL/6 (Jax stock #000664) for over 30 generations (Jax stock #004302; C57BL/6 background) or to *Zp3* ^*Cre*^ mice (C57BL/6‐Tg(Zp3‐cre)93Knw/J, Jax stock #003651). The *HA‐Trim21* global‐ and oocyte‐specific overexpression mice were then crossed to Hsd:NSA(CF‐1) for two generations, then bred to homozygosity. To maintain the colony, there are two sets of breedings: OE/OE;*Zp3* are kept as homozygous pairs, and *Trim21/Trim21*;Zp3/+ males are crossed to *Trim21/Trim21* females. Neither set of breeding pairs has any issues with fertility and they have normal size litters. These mice will be deposited at the Mutant Mouse Regional Resource Centers (MMRRC) upon acceptance of the manuscript.

### 3.3 Mouse Oocyte Collection, Culture, and Microinjection

The media for oocyte and egg collection and microinjection was HEPES‐buffered MEMα (Mehlmann, Uliasz, and Lowther 2019). Isolated GV‐stage oocytes were collected after popping ovaries in culture medium with a 30 gauge needle, and cumulus cells were removed using a small‐bore glass pipet. When collecting oocytes, 5–10 μM milrinone was included in the medium to prevent spontaneous meiotic resumption. For overnight culture, oocytes were washed into bicarbonate‐buffered MEMα (Mehlmann, Uliasz, and Lowther 2019) without milrinone and supplemented with 5% fetal bovine serum (FBS). Oocytes were cultured overnight at 37°C in a humidified incubator with 5% CO_2_/95% air. MII‐stage eggs were collected from superovulated mice following injection of 5–10 IU (depending on age and weight) eCG and 5–10 IU hCG. Eggs were released from swollen ampullae ~13–16 h following injection of hCG and cumulus cells were dispersed with 0.3 mg/mL hyaluronidase (Type IV‐S) made in HEPES‐buffered MEMα. For experiments in which levels of HA‐TRIM21 were compared in oocytes and eggs, cumulus masses from superovulated mice were first released into hyaluronidase‐containing MEMα, and then ovaries were transferred to MEMα containing milrinone to collect oocytes from the ovaries.

Quantitative microinjection was performed as previously described (Kline 2009; Lowther et al. 2009) using calibrated, beveled glass micropipets backfilled with mercury. Oocytes and eggs were microinjected with ~10 pL of antibody solution that was ~0.5–1 mg/mL, for a final injected concentration of 25–50 μg/mL. Calculations of injected concentrations were based on a volume of 200 pL. Oocytes and eggs that lysed during injection were discarded.

Affinity‐purified IP_3_ receptor antibody was obtained from James Watras (UConn Health; (Runft, Watras, and Jaffe 1999)) and SNAP23 antibody was from ThermoFisher (#PA1‐738). Prior to microinjection, antibodies were spin‐dialyzed into PBS and concentrated using either Amicon Ultra‐0.5 mL, 10 kDa cutoff or Vivaspin 500, 50 kDa cutoff centrifugal filters (Millipore). Concentrated antibodies were diluted to 1 mg/mL in PBS containing 0.05% NP‐40 to reduce stickiness. To prepare mRNA, a gBlock fragment (IDT) containing the HA‐TRIM21 coding sequence with appropriate unique restriction sites (SalI‐AsiSI on 5′ end and AsiSI‐SpeI on 3′ end) was inserted it into the pSK vector. The *HA‐Trim21‐pSK* vector was linearized with NotI and used as a template for in vitro transcription.

### 3.4 Preparation of Cell/Tissue Lysates and Western Blotting

Following microinjection, oocytes and eggs were incubated on a warm stage at 37°C and were collected ~3 or 4 h later, or after overnight incubation. Cells were counted and transferred to tubes in a small volume of medium (1–2 μL), 15 μL of 1× Laemmli buffer was added, and the samples were frozen at −20°C until use.

Brain, liver, and kidney samples were collected and washed in 2 mL ice‐cold PBS. Tissues were minced with scissors, then were sonicated in 1 mL RIPA buffer (Teknova) containing protease inhibitor cocktail (Calbiochem). Samples were centrifuged at 800×*g* and the supernatants stored at −80°C. To obtain granulosa cells, ovaries were popped with a 30 gauge syringe needle in culture medium and the released granulosa cells were collected and transferred to a centrifuge tube. Cells were centrifuged briefly and the pellet was washed with PBS, centrifuged again, and the cells were resuspended in 25 μL RIPA containing protease inhibitors. Protein concentrations were determined using a BCA assay (Pierce).

Western blotting was performed as previously described (Mehlmann, Uliasz, and Lowther 2019). Briefly, proteins were separated in 4%–20% acrylamide gels (Bio‐Rad, Hercules, CA) and then electrophoretically transferred to PVDF membranes. Membranes were blocked using Advansta blocking reagent (Advansta) and were incubated in primary antibodies overnight at 4°C. IP_3_ receptor and SNAP23 antibodies were the same as the ones injected, ZP2 antibody was obtained from Jurrien Dean (NIH) (Burkart et al. 2012), RAB3A antibody was from LS‐Bio (# LSC‐802955), and HA antibody was from Cell Signaling (#3742). Following washing and incubating with horseradish peroxidase‐conjugated secondary antibodies, blots were developed using Western‐Bright Sirius horseradish peroxidase substrate (Advansta), using a charge‐coupled device camera (G: box Chemi XT4; Syngene [Frederick, MD]). Densitometric analyses were done using ImageJ software.

### 3.5 Quantitative RT‐PCR

RNA from 40 GV oocytes and 40 in vitro matured MII eggs collected from the same *Zp3* ^*Cre*^ mouse was isolated using the RNAqueous Micro total RNA Isolation kit (Thermo scientifc) and genomic DNA was removed by DNase treatment. cDNA was synthesized using iScript (Bio‐Rad) and one oocyte equivalent per well was used for qPCR using SsoAdvanced Sybr green supermix (Bio‐Rad) on a Cfx Connect real‐time system (Bio‐Rad) and assayed in duplicate. The 2^−ΔΔCt^ (cycle threshold) method was used to calculate relative expression levels, which are reported as a fold change. The primers used for *Trim21* were F 5′‐ACCCATACGATGTTCCAGATT‐3′ and R 5′‐ATTCGATACTCATAGGCTCCAC‐3′.

### 3.6 Preparation and Electroporation of Neural Cell Cultures

Newborn mice (0 days old) were anesthetized with isoflurane and decapitated. Brains were removed and isolated cortical tissue was finely minced with forceps, then transferred into dissection medium (27.8 μM D‐glucose, 20.5 μM sucrose, and 20 μM HEPES in Hank's Balanced Salt Solution (HBSS) without calcium, magnesium, or bicarbonate (Cellgro) and containing 0.025% trypsin (Gibco 25,200‐056)). Cortical tissue was incubated in trypsin‐containing medium for 20–30 min in a 37°C water bath and dissociated cells were spun at ~720×*g* for 3 min at room temperature. Cells were resuspended and gently pipetted up and down in 1 mL plating medium (Neural Basal Medium, Gibco #21103–049; 1× Pen/Strep, Gibco #15140–122; 1× L‐Glutamine, Gibco #25030–081; and 10% heat‐inactivated horse serum, Gibco #26050–088) using flame‐polished Pasteur pipets and plated in six‐well dishes at a density of one pup per well. After 3 days in culture, nonadherent clumps of cells were collected and suspended in 0.25% trypsin–EDTA for 5 min and replated to wells with adherent cells. Cells were then grown to confluence (8–11 days), trypsinized to dissociate them from plates, divided into equal groups, and electroporated with a Neon Transfection System electroporator in Buffer R, according to the manufacturer's instructions, using two pulses, 20 msec each, 1400 V. Following electroporation, cells were transferred back to plating medium and incubated for 4 h in a humidified 37°C incubator with 5% CO_2_/95% air. Cells were pelleted, washed once with PBS, resuspended in 15 μL 1× Laemmli sample buffer, and the entire sample was run per lane in a polyacrylamide gel. Western blotting was performed as outlined above, with the exception that blots were stained with Revert 700 Total Protein Stain (LICORbio) and imaged prior to the blocking step. For these experiments, densitometric band values were normalized to total protein.

### 3.7 Statistical Analysis

Data were analyzed using paired *t*‐tests and one‐way ANOVA, as indicated in the figure legends. Statistical tests were performed using GraphPad Prism. *p* < 0.05 was considered to be significant.

## Ethics Statement

All animal experiments were approved by the Institutional Animal Care and Use Committee at UConn Health.

## Data Availability

The data that support the findings of this study are available from the corresponding author upon reasonable request.

## References

[dvg23616-bib-0001] Burkart, A. D. , B. Xiong , B. Baibakov , M. Jimenez‐Movilla , and J. Dean . 2012. “Ovastacin, a Cortical Granule Protease, Cleaves ZP2 in the Zona Pellucida to Prevent Polyspermy.” Journal of Cell Biology 197, no. 1: 37–44. http://www.ncbi.nlm.nih.gov/entrez/query.fcgi?cmd=Retrieve&db=PubMed&dopt=Citation&list_uids=22472438. 22472438 10.1083/jcb.201112094 PMC3317803

[dvg23616-bib-0002] Chu, V. T. , T. Weber , R. Graf , et al. 2016. “Efficient Generation of *Rosa26* Knock‐In Mice Using CRISPR/Cas9 in C57BL/6 Zygotes.” BMC Biotechnology 16: 4. 10.1186/s12896-016-0234-4. 26772810 PMC4715285

[dvg23616-bib-0003] Clift, D. , W. A. McEwan , L. I. Labzin , et al. 2017. “A Method for the Acute and Rapid Degradation of Endogenous Proteins.” Cell 171, no. 7: 1692–1706.e18. 10.1016/j.cell.2017.10.033. 29153837 PMC5733393

[dvg23616-bib-0004] de Vries, W. N. , L. T. Binns , K. S. Fancher , et al. 2000. “Expression of *Cre* Recombinase in Mouse Oocytes: A Means to Study Maternal Effect Genes.” Genesis 26. no. 2: 110–112. https://www.ncbi.nlm.nih.gov/pubmed/10686600. 10686600

[dvg23616-bib-0005] Fischer von Mollard, G. , B. Stahl , C. Li , T. C. Sudhof , and R. Jahn . 1994. “Rab Proteins in Regulated Exocytosis.” Trends in Biochemical Sciences 19, no. 4: 164–168. 10.1016/0968-0004(94)90278-x. 8016866

[dvg23616-bib-0006] Hrdlicka, H. C. , R. C. Pereira , B. Shin , et al. 2021. “Inhibition of miR‐29‐3p Isoforms via Tough Decoy Suppresses Osteoblast Function in Homeostasis but Promotes Intermittent Parathyroid Hormone‐Induced Bone Anabolism.” Bone 143: 115779. 10.1016/j.bone.2020.115779. 33253931 PMC7770763

[dvg23616-bib-0007] Jentoft, I. M. A. , F. J. B. Bauerlein , L. M. Welp , et al. 2023. “Mammalian Oocytes Store Proteins for the Early Embryo on Cytoplasmic Lattices.” Cell 186, no. 24: 5308–5327 e25. 10.1016/j.cell.2023.10.003. 37922900

[dvg23616-bib-0008] Kline, D. 2009. “Quantitative Microinjection of Mouse Oocytes and Eggs.” Methods in Molecular Biology 518: 135–156. 10.1007/978-1-59745-202-1_11. 19085140

[dvg23616-bib-0009] Lacham‐Kaplan, O. , and A. Trounson . 2008. “Reduced Developmental Competence of Immature, In‐Vitro Matured and Postovulatory Aged Mouse Oocytes Following IVF and ICSI.” Reproductive Biology and Endocrinology 6: 58. 10.1186/1477-7827-6-58. 19040764 PMC2636812

[dvg23616-bib-0010] Lowther, K. M. , V. N. Weitzman , D. Maier , and L. M. Mehlmann . 2009. “Maturation, Fertilization, and the Structure and Function of the Endoplasmic Reticulum in Cryopreserved Mouse Oocytes.” Biology of Reproduction 81. no. 1: 147–154. http://www.ncbi.nlm.nih.gov/entrez/query.fcgi?cmd=Retrieve&db=PubMed&dopt=Citation&list_uids=19299317. 19299317 10.1095/biolreprod.108.072538 PMC3093990

[dvg23616-bib-0011] Madisen, L. , T. A. Zwingman , S. M. Sunkin , et al. 2010. “A Robust and High‐Throughput *Cre* Reporting and Characterization System for the Whole Mouse Brain.” Nature Neuroscience 13, no. 1: 133–140. 10.1038/nn.2467. 20023653 PMC2840225

[dvg23616-bib-0012] Mallery, D. L. , W. A. McEwan , S. R. Bidgood , G. J. Towers , C. M. Johnson , and L. C. James . 2010. “Antibodies Mediate Intracellular Immunity Through Tripartite Motif‐Containing 21 (TRIM21).” Proceedings of the National Academy of Sciences of the United States of America 107, no. 46: 19985–19990. 10.1073/pnas.1014074107. 21045130 PMC2993423

[dvg23616-bib-0013] Mehlmann, L. M. 2005. “Oocyte‐Specific Expression of *Gpr3* Is Required for the Maintenance of Meiotic Arrest in Mouse Oocytes.” Developmental Biology 288. no. 2: 397–404. http://www.ncbi.nlm.nih.gov/entrez/query.fcgi?cmd=Retrieve&db=PubMed&dopt=Citation&list_uids=16289135. 16289135 10.1016/j.ydbio.2005.09.030 PMC1868506

[dvg23616-bib-0014] Mehlmann, L. M. , and D. Kline . 1994. “Regulation of Intracellular Calcium in the Mouse Egg: Calcium Release in Response to Sperm or Inositol Trisphosphate Is Enhanced After Meiotic Maturation.” Biology of Reproduction 51, no. 6: 1088–1098. http://www.ncbi.nlm.nih.gov/entrez/query.fcgi?cmd=Retrieve&db=PubMed&dopt=Citation&list_uids=7888488. 7888488 10.1095/biolreprod51.6.1088

[dvg23616-bib-0015] Mehlmann, L. M. , K. Mikoshiba , and D. Kline . 1996. “Redistribution and Increase in Cortical Inositol 1,4,5‐Trisphosphate Receptors After Meiotic Maturation of the Mouse Oocyte.” Developmental Biology 180, no. 2: 489–498. http://www.ncbi.nlm.nih.gov/entrez/query.fcgi?cmd=Retrieve&db=PubMed&dopt=Citation&list_uids=8954721. 8954721 10.1006/dbio.1996.0322

[dvg23616-bib-0016] Mehlmann, L. M. , T. F. Uliasz , and K. M. Lowther . 2019. “SNAP23 Is Required for Constitutive and Regulated Exocytosis in Mouse Oocytesdagger.” Biology of Reproduction 101, no. 2: 338–346. 10.1093/biolre/ioz106. 31201423 PMC6736185

[dvg23616-bib-0017] Niwa, H. , K. Yamamura , and J. Miyazaki . 1991. “Efficient Selection for High‐Expression Transfectants With a Novel Eukaryotic Vector.” Gene 108, no. 2: 193–199. 10.1016/0378-1119(91)90434-d. 1660837

[dvg23616-bib-0018] Powell, J. E. , C. K. W. Lim , R. Krishnan , et al. 2022. “Targeted Gene Silencing in the Nervous System With CRISPR‐Cas13.” Science Advances 8, no. 3: eabk2485. 10.1126/sciadv.abk2485. 35044815 PMC8769545

[dvg23616-bib-0019] Runft, L. L. , J. Watras , and L. A. Jaffe . 1999. “Calcium Release at Fertilization of *Xenopus* Eggs Requires Type I IP_3_ Receptors, but Not SH2 Domain‐Mediated Activation of PLCγ or G_q_‐Mediated Activation of PLCβ.” Developmental Biology 214, no. 2: 399–411. 10.1006/dbio.1999.9415. 10525343

[dvg23616-bib-0020] Tang, S. H. , F. J. Silva , W. M. Tsark , and J. R. Mann . 2002. “A Cre/loxP‐Deleter Transgenic Line in Mouse Strain 129S1/SvImJ.” Genesis 32, no. 3: 199–202. 10.1002/gene.10030. 11892008

[dvg23616-bib-0021] Yoshimi, R. , T. H. Chang , H. Wang , T. Atsumi , H. C. Morse 3rd , and K. Ozato . 2009. “Gene Disruption Study Reveals a Nonredundant Role for TRIM21/Ro52 in NF‐kappaΒ‐Dependent Cytokine Expression in Fibroblasts.” Journal of Immunology 182, no. 12: 7527–7538. 10.4049/jimmunol.0804121. PMC2803686 19494276

